# Case Report: SARS-CoV-2 Infection in a Child With Suprasellar Tumor and Hypothalamic-Pituitary Failure

**DOI:** 10.3389/fendo.2021.596654

**Published:** 2021-03-16

**Authors:** Rossella Gaudino, Valentina Orlandi, Paolo Cavarzere, Matteo Chinello, Franco Antoniazzi, Simone Cesaro, Giorgio Piacentini

**Affiliations:** ^1^ Section of Paediatrics, Department of Surgery, Dentistry, Paediatrics and Gynaecology, University of Verona, Verona, Italy; ^2^ Endocrinology Unit, Section of Paediatrics, Department of Surgery, Dentistry, Paediatrics and Gynaecology, University of Verona, Verona, Italy; ^3^ Pediatric Hematology Oncology, Department of Mother and Child, Azienda Ospedaliera universitaria Integrata, Verona, Italy

**Keywords:** Sars-CoV2, COVID-19, GNS germ cell tumor, pediatric endocrinology, diabetes insipidus, hypothalamic-pituitary failure

## Abstract

In early 2020, a novel coronavirus leading to potentially death was discovered. Since then, the 2019 coronavirus disease (COVID-19) has spread to become a worldwide pandemic. Beyond the risks strictly related to the infection, concerns have been expressed for the endocrinological impact that COVID-19 may have, especially in vulnerable individuals with pre-existing endocrinological health conditions. To date new information is emerging regarding severe acute respiratory syndrome-related coronavirus 2 (SARS-CoV-2) in children but the literature is still scarce concerning this infection in patients with intracranial malignant neoplasms. We report a 9-year-old child infected with SARS-CoV-2 and recent diagnosis of suprasellar non-germinomatous germ cell tumor also suffering from diabetes insipidus and hypothalamic-pituitary failure (hypothyroidism, adrenal insufficiency, hypothalamic obesity and growth hormone deficiency) and its clinical course. The patient remained asymptomatic for the duration of the infection without requiring any change in the replacement therapeutic dosages taken before the infection. We then discuss the proposed approach to treat a pediatric patient with SARS-CoV-2 infection and hypothalamic-pituitary failure and we include a review of the literature. Our report suggests that SARS-CoV-2 infection is usually mild and self-limiting in children even those immunocompromised and with multiple endocrinological deficits. Patients are advised to keep any scheduled appointments unless informed otherwise.

## Introduction

The novel severe acute respiratory syndrome coronavirus 2 (SARS-CoV-2), which first appeared in December 2019 in Wuhan, has caused the coronavirus disease 2019 (COVID-19) related pandemic and has been declared a significant threat to international health by the World Health Organization.

Based on data from large studies, obesity emerged as a strong and independent determinant of increased risk of morbidity and mortality in adult patients infected with SARS- CoV-2 (1) and patients with cancer have a higher risk of worse outcomes with increased risk of severity and mortality. As in adults, but less frequently, children with comorbidities as malignancies, diabetes, obesity and immune disorders, are more likely to develop severe conditions from COVID-19 (2).

Management of patients with pituitary tumors and their associated hormonal abnormalities and mass effect complications can be challenging even during normal times and need to be preferably handled in Pituitary Centers of Excellence, with a multidisciplinary approach, especially in complex cases. Some of the diagnostic and management dilemmas regarding adult patients with pituitary tumors, and guidance on safe and as effective as possible delivery of care in the COVID-19 era, are have recently been reported (3). Indeed, evidence suggests that SARS-CoV-2 infection in children with oncological comorbid and endocrinological health problems may be challenging in terms of management and could require complex care.

## Case Description

We report the case of a 9-year-old Caucasian female affected by suprasellar non-germinomatous germ cell tumor (NGGCT) associated with multiple pituitary deficit, who tested positive for SARS-CoV-2 an April 1, 2020.

**Figure 1:**
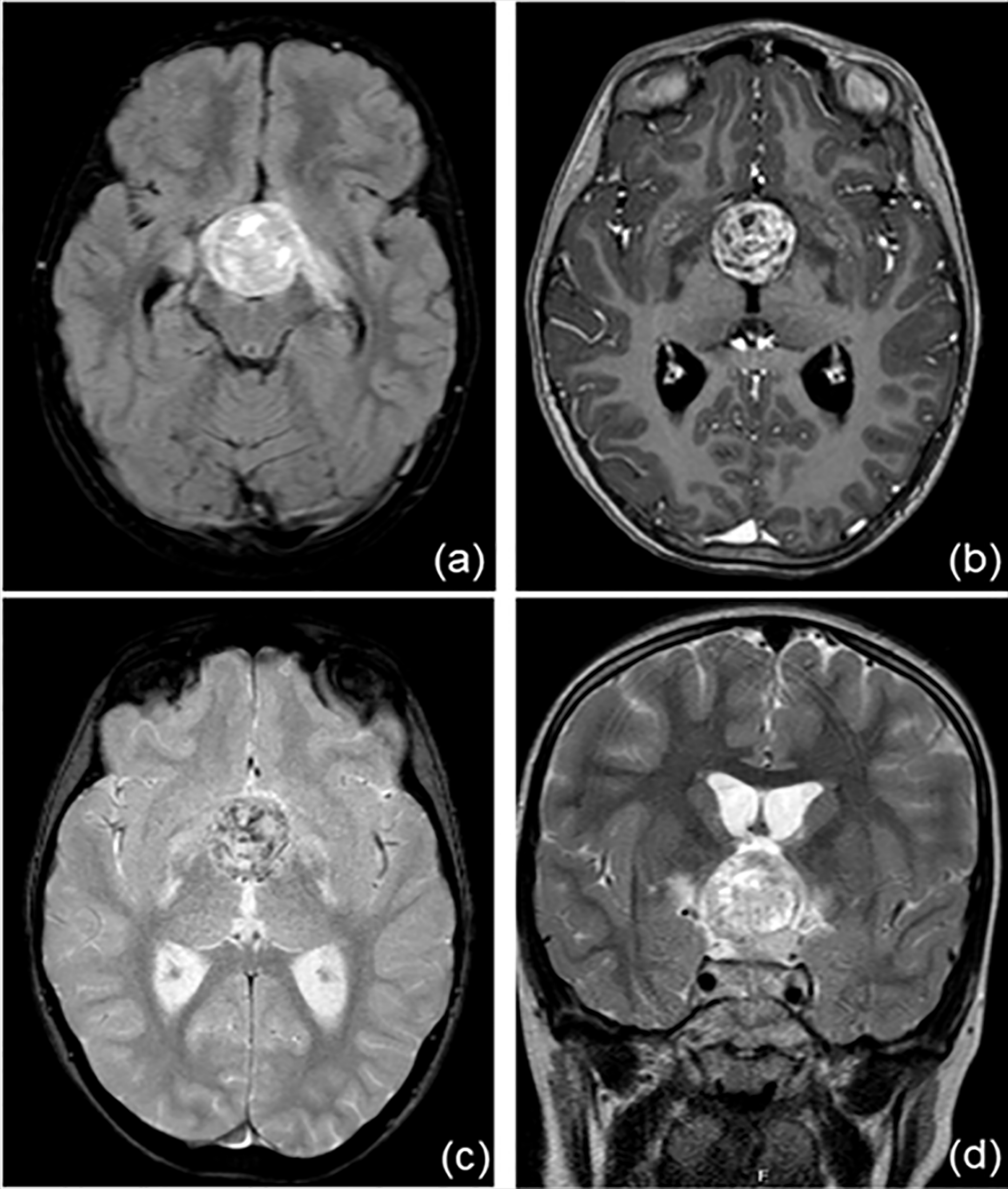
Brain magnetic resonance imaging at the time of diagnosis. There is a recognizable, voluminous expansive lesion at the hypothalamic level (**A**, FLAIR sequence) with an inhomogeneous structure, first read as calcifications (**B**, T1 sequence; **C**, T2 sequence). Optic chiasma, hypothalamic-pineal peduncle and mammillary body are not recognizable. (**D**, T2W_TSE sequence).

Her problem began 2 years earlier, with polyuria, polydipsia (she drank almost 8 liters of water/day) and growth delay. In October 2019, she came to Verona’s Paediatric Emergency Department in a state of confusion. Brain magnetic resonance imaging revealed a suprasellar mass, compatible with germinoma (**Figure 1**). This hypothesis was confirmed by high levels of alfa-fetoprotein and human chorionic gonadotropin (hCG) in plasma and cerebrospinal fluid (CFS). Based on alfa-fetoprotein and hCG levels the tumor was classified as a non germinomatous germ cell tumor (NGGCT). Ophthalmologic evaluation and a study of visual fields were normal. At the time of diagnosis, blood exams tests confirmed a multiple pituitary deficit with diabetes insipidus, central hypothyroidism, hypocortisolism, growth hormone deficiency and mild hyperprolactinemia. The patient’s laboratory test results at time of diagnosis are shown in **Table 1**. The girl started therapy with levothyroxine (initially at dosage 25 mcg/day) and desmopressin (initially intranasal, then sublingual). She was given dexamethasone after the MRI to reduce tumoral edema. Over the next few days the patient was found to have “cerebral salt wasting” with hyponatremia, high urinary sodium excretion (129 mmol/L) and persistent polyuria, so we started fludrocortisone. It was stopped after four days when laboratory tests normalized. She continued therapy with desmopressin, levothyroxine and hydrocortisone and the patient had periodical laboratory tests and therapeutic adjustments.

**Table 1:** Laboratory tests and physical examination at the time of diagnosis.

Laboratory test		Normal values
Copeptin (pmol/L)	<0,9	1.5-12.0
ACTH (pg/mL)	<0.84	1.8-13.2
P-osmolality (mOsm/kgH2O)	292	275-295
Na (mmol/L)	147	135-145
Cortisol (nmol/L)	25	133-537
HPRL (ug/L)	35	4-23
IGF1 (ng/mL)	52,85	68,84-230
TSH (mUI/L)	0,41	0.30-4.20
fT4 (pmol/L)	6,7	11.0-22.0
U-osmolality (mOsm/kgH2O)	92	50-1200
U-Na (mmol/L)	29	
alfa-fetoprotein (plasma) (ng/mL)	589.83	1,09-8,04
alfa-fetoprotein (liquor) (Index)	303	<1
hCG (plasma) (IU/L)	815	<5
hCG (liquor) (Index)	12	<9
Physical examination		Centiles (Cacciari’s charts)
Weight (kg)	23	16,01°
Height (cm)	119	8,49°
BMI (kg/m^2^)	16,24	39,04°

In October 2019 chemotherapy according to SIOP CNS GCT II standard risk protocol for non-germinomatous germ cell tumor (NGGCT) was started. The first cycle was complicated by hydrocephalus that required ventricular-peritoneal derivation. At the end of the third cycle, the MRI showed a partial response to chemotherapy. Therefore, in January 2020, the patient underwent neurosurgery. The histopathological examination was conclusive for immature teratoma. Follow-up MRI one month after surgery showed a suspected small residual lesion. Chemotherapy with high-dose etoposide was started.

From the 8 March, the “lockdown” for all Italy was adopted together with the use of surgical mask, hand hygiene, and social distancing, to prevent the massive spread of SARS-CoV-2; moreover, a screening program by nasopharyngeal swab (NPS) for any patient and caregiver parent accessing the Pediatric Hematology Oncology Unit was started (4). On March 12, the patient (and her mother) underwent the NPS and both resulted negative for SARS-CoV-2. She was taking desmopressin (sublingual tablets 60 mcg, 2 + 1+2, 300 mcg/day), levothyroxine at 75 mcg/day and hydrocortisone at 8,92 mg/m^2.^day divided into 2 doses. Therapy with recombinant GH was not yet started. Since her neurosurgical operation, she suffered from hypothalamic obesity (incontrollable hyperphagia, decreased satiety and decreased physical activity) with massive weight gain.

On March 5 she was admitted to the Pediatric Oncology Unit for mobilization chemotherapy with high-dose etoposide and granulocyte colony-stimulating factor (G-CSF) followed by peripheral stem cell collection for autologous stem-cell transplantation (ASCT). On April 1 she was admitted to the Pediatric Oncology Unit for ASCT. Her weight was 40,9 kg (93,21 percentiles based on Cacciari’s charts) and her height was 120,2 cm (6,08 percentiles based on Cacciari’s charts), with a BMI of 28,31 kg/m^2^ (99,06° centiles based on Cacciari’s charts).

When she came for screening, a second NPS for SARS-CoV-2 was performed on the patient and parent caregiver (the father) and both resulted positive. The pre-transplant conditioning regimen (HD PEI cycle - cisplatin, etoposide and ifosfamide- according to SIOP CNS GCT II protocol) was already started on April 2 in the morning, and was therefore withdrawn as soon as the result of NPS was communicated in the evening of the same day (administration of day 1 of conditioning regimen). Both patient and father were quarantined in a pediatric COVID19 section of the department to monitor the patient’s symptoms or signs of SARS-CoV-2 infection. During observation, blood count, urine tests. inflammatory markers, chest X-ray were unremarkable. In particular, her blood exams showed leukocytes 6,87x10^9/L, neutrophils 5,77x10^9/L and lymphocytes 0,40x10^9/L. No respiratory, cutaneous signs, or fever appeared during recovery and the patient continued her previous therapies. The most significant laboratory tests before, during and after Sars-Cov2 infection are summarized in **Table 2**. Her diuresis remained in the range of 2-2,9 ml/kg/h. The patient was discharged 3 days later, in good general conditions. Her weight at discharge was stable. Another two nasal and pharyngeal swabs were both negative after 28 days (**Table 3**). The ASCT was performed on April 30 after a conditioning regimen with high dose ifosfamide, etoposide and cisplatin. She received 4.38 x 10^6^/kg CD34+. The patient engrafted on day + 9 for neutrophil and on day + 12 for platelets and was discharge home in good clinical conditions. In accordance with Verona University and University of Verona Institutional review Board (IRB) policies, case reports are not considered to be research subject to IRB review and are thus except from formal IRB approval for publication. Written informed consent was obtained from the patient’s legal guardians for the publication of any potentially identifiable images or data included in this article.

**Table 2:** Laboratory tests before, during and after Sars-Cov2 infection.

	P-osmolality (mOsm/KgH2O)	P-Na (mmol/L)	U-Na (mmol/L)	U-osmolality (mOsm/KgH2O)	PCR (mg/L)	TSH (mUI/L)	fT4 (pmol/L)
7 days before	290	145					
Day 0 (8:00 am)	286	143	96	235			
Day 0 (4:00 pm)	298	145	36	95			
Day +1 (00:30 am)	286	140					
Day +1 (8:00 am)	293	143	<20	68	4	<0,005	16,3
Day +2 (6:00 am)	291	142	189	585			
Day + 3 (8:00 am)	283	138	143	587			
Day + 20(I negative swab)							
Day + 28 (II negative swab)	280	136	124	718			

**Table 3:** Nasopharyngeal swabs with cycle threshold (Ct) value results.

Date	Result	Ct value S gene (FAM)	Ct value OFR1ab (JOE)	Ct value RNA IC (Q670)
03/12/2020	Not detectable			
04/02/2020	Detectable	27	27.1	29.1
04/22/2020	Not detectable			
04/30/2020	Not detectable			

## Discussion

Our case report, for the first time, describes the evolution of SARS-CoV-2 infection in a child with intracranial malignant neoplasms and multiple pituitary failure. Clinical features of SARS-CoV-2 infection in adults have been reported elsewhere. Compared to adult patients, reported cases of pediatric patients had clinically milder symptoms and showed fewer alterations in radiological and laboratory testing parameters. A systematic review confirmed that Chinese pediatric patients with COVID-19 had a good prognosis and recovered within 1 to 2 weeks after disease onset, and no cases of pediatric death from COVID-19 were reported in the age range of 0 to 9 years. One death was reported in the 10 to 19 years range, but no further information was provided on this patient (5).

Primary central nervous system (CNS) germ cell tumors (GCT) are a heterogeneous group of tumors that are still poorly understood. Most of these tumors develop along the midline, mainly from the pineal gland, followed by tumors arising in the suprasellar cistern (6). The suprasellar region is the second most common location of intracranial germinomas and it has been clear for decades that suprasellar germinomas can cause diabetes insipidus, visual disturbances, and pituitary dysfunctions. Treatment protocols for CNS GCTs have evolved greatly and the survival rate for germinomas is more than 90%, whereas NGGCTs have a 10-year overall survival of 60-80% (7). In most cases, these tumors are associated with permanent endocrine disorders that must be continuously followed up (6).

Our patient was affected by non-germinomatous germ cell tumor (NGGCT), based on alfa-fetoprotein, hCG levels in plasma and CSF and based on histopathological examination. The girl had secondary adrenal insufficiency, diabetes insipidus, central hypothyroidism, hypothalamic obesity and GH deficiency.

How to manage patients with pituitary tumors without easy access to full investigations in the COVID-19 era was described by Fleseriu et al. (3) and the response to COVID-19 by endocrinologists and diabetologists has been described (8). There is currently no proven concern that pituitary tumors per se affect the immune system, apart from corticotropic adenomas causing cortisol excess. Patients with uncontrolled Cushing’s disease are at higher risk of infections, which affects their mortality risk (9). Moreover, those patients may be taking supraphysiologic doses of glucocorticoids and might be more susceptible to COVID-19 as a result of the immunosuppressive effects of steroids.

Our patient had long suspended the high antiedema doses of dexamethasone at the time of infection and she was taking replacement doses of hydrocortisone. We presumed that she was infected through contact with her father at home, and she tested positive regardless of the therapies she was taking.

The impact of COVID-19 on patients with pituitary-neuroendocrine disease also needs to be considered. Included in the management of a patient with hypopituitarism are replacement of hormone deficiencies, regular screening for the development of new pituitary hormone deficiencies, surveillance of the underlying cause (usually a tumor). Many of these patients have hypopituitarism including secondary adrenal insufficiency, requiring stress dose glucocorticoid supplementation in adult patients and in children (10). Also, patients with adrenal insufficiency are at increased risk of infection (11), which may be complicated by developing an adrenal crisis. However, there is currently no evidence that adrenal insufficiency patients are more likely to develop a severe course of the viral disease. Adrenal crisis occurs when cortisol availability is reduced or low at a time of increased need for cortisol, such as during a severe infection (e.g. influenza or viral respiratory infection). Therefore, to prevent adrenal crisis patients are instructed to immediately double or triple their daily dose of hydrocortisone during an illness, whereas the dose adjustment during a stress not related to illness.

Our young patient never showed signs or symptoms indicating clinical deterioration, which in patients affected by COVID-19, typically occur 7-10 days after the onset of the first COVID-associated symptoms. Our report confirms that an asymptomatic child, who tested SARS-CoV-2 positive, for example through family or oncology screening, does not need to increase their routine hydrocortisone replacement dose. However, we stress that, in case of an acute COVID-19 infection with clinical deterioration, children with adrenal insufficiency should immediately receive a parenteral injection of 50-100 mg/m2 hydrocortisone (usually 25 mg for infants, 50 mg for school children) (12).

No published reports so far have reported a higher prevalence of dysnatremia in COVID-19. However, the COVID-19 pandemic has limited the accessibility of blood testing, and the priority of routine treatment of central DI should be to avoid hyponatremia. It emphasizes the importance of delaying desmopressin doses, to allow regular periods of free water clearance, so that excess water intake does not lead to dilutional hyponatremia (13). Our patient had adipsic DI and hypothalamic obesity, and there is accumulating evidence that obesity per se may be an important risk factor for poor outcome in patients with Covid-19 (1). Therefore, our patient could have been among the most vulnerable; she had a very close follow-up for the superimposed illness without, however, manifesting imbalance.

Data on thyroid function or thyroid pathology are not yet available in COVID-19. Patients with underlying hypothyroidism or hyperthyroidism are advised to continue their prescribed medications as usual (14). Our patient, with central hypothyroidism, in agreement with the British Thyroid Association and the Society for Endocrinology (BTA/SfE) consensus statement, did not change the usual dose of levothyroxine during the SARS-CoV-2 infection. Due to the recent diagnosis, despite her GH deficiency, our patient at the time of the SARS-CoV-2 infection had not yet started treatment. Although infections can cause GH deficiency (15), there is no data in the literature on the increased risk of infections in patients with GH deficiency. Critical illness induced a rise in GH and IGFBP1 and a fall in IGF-I and IGFBP3, as previously described. Unfortunately, during the SARS-CoV-2 infection, we did not perform our patient’s the dosage of GH or IGF-1 considered in the emergency to be of secondary importance.

Finally, the first systematic review dealing with the impact of immunosuppression on SARS-CoV-2 infection affirmed that immunosuppressed hosts may not present a greater risk of an increased severity of disease, compared to the general population (16). Considering instead, the evidence available to date on SARS-CoV-2 infection outcomes in patients with immunosuppression (either due to their disease or the use of immunosuppressants) its behavior is not clear. The patients with cancer and recent treatment of cancer (chemotherapy or surgery) have a higher risk of worse outcomes, with faster deterioration than those without cancer, an increased risk of severity and mortality having been shown through two meta-analyses. This could perhaps be explained in that the severity of SARS-CoV-2 infection has been associated with an aberrant inflammatory response (cytokine storm) (17). To increase our knowledge in disease mechanisms, we need to learn from clinical and immunologic characteristics of patients with severe in contrast to moderate disease. We interrupted the conditioning regimen in our patient because at the time of diagnosis of infection there was no consensus about chemotherapy in similar patients SARS-CoV-2 positive. In addition, her ASCT was not considered urgent, so it was reasonable to delay the treatment after the negativity of NPS. For these reasons, the strength of our description is the need to inform the scientific community about this case report. The major merits were detecting novelties, pharmacovigilance and high applicability when other research designs were difficult to carry out. The major limitations were the lack of ability to generalize and the retrospective design.

## Management Recommendations

During this COVID-19 pandemic, in children with hypopituitarism, proper methods for viral respiratory infection control and prevention, supportive therapies, and considerations of potential therapies relevant to the pituitary disorders and any underlying comorbidities should be undertaken. The caregivers are urged to request for COVID-19 testing for patients if exposed. Even if symptoms are mild or if fever is present contacting the team (the physicians or nurse by telephone, e-mail, or telemedicine) is mandatory in order to seek medical guidance. Maintain of adequate hydration, adhere to daily medications, and adapt to increased replacement dosages if clinically indicated, is recommended.

## Patient Perspective

Recognizing SARS-CoV-2 as a possible causal trigger of endocrinological failure and its exacerbation is of particular importance to improving its early diagnosis. In addition, the aim of our report is to be informative while reassuring at the same time. Our case suggests that SARS-CoV-2-infection is usually mild and self-limiting in children even in those who are immunocompromised. Patients are advised to keep any scheduled appointments unless informed otherwise.

## Data Availability Statement

The original contributions presented in the study are included in the article/supplementary material. Further inquiries can be directed to the corresponding author.

## Ethics Statement

Ethical review and approval was not required for the study on human participants in accordance with the local legislation and institutional requirements. Written informed consent to participate in this study was provided by the participants’ legal guardian/next of kin.

## Author Contributions

RG designed the study. VO, MC, and PC collected data. RG and PC performed data interpretation. RG and VO wrote the first draft of the manuscript. RG, SC, AF, and GP critically reviewed the manuscript for intellectual content. All authors contributed to the article and approved the submitted version.

## Conflict of Interest

The authors declare that the research was conducted in the absence of any commercial or financial relationships that could be construed as a potential conflict of interest.
